# Back mobility and interincisor distance ranges in racially diverse North American healthy children and relationship to generalized hypermobility

**DOI:** 10.1186/1546-0096-10-17

**Published:** 2012-06-20

**Authors:** Sophie L Woolston, Timothy Beukelman, David D Sherry

**Affiliations:** 1 Department of Medicine, Hospital of the University of Pennsylvania, 3400 Spruce St., Philadelphia, PA, 19104, USA; 2 Department of Pediatrics, Rheumatology, University of Alabama-Birmingham, 16000 7th Ave. S., Birmingham, AL, 35233, USA; 3 Department of Pediatrics, Rheumatology, The Children’s Hospital of Philadelphia, 34th and Civic Center Blvd., Philadelphia, PA, 19104, USA

**Keywords:** “Temporomandibular joint”, “Arthritis, juvenile Rheumatoid”, “Reference values”, “Back ph [Physiology]”, “Child” “Joint instability”

## Abstract

**Background:**

Given the dearth of normal values, we conducted a cross-sectional study of North American racially diverse children to determine normal values of interincisor distance and lower spine flexion.

**Methods:**

Demographs of 307 children aged 5–17 seeking treatment emergency care were obtained along with interincisor distance measured by incisor tooth-to-tooth gap, lower spine flexion measured by the Schober and modified Schober measurements, popliteal extension, hypermobility (Beighton) score, weight and height.

**Results:**

Normal range of motion values for the Schober was a mean of 14.3 cm (95% confidence interval (CI) was 11.2 to 17. cm) and the mean modified Schober’s was 21.6 cm (95% CI 18.4 cm to 24.8 cm). Retained lumbar lordosis on forward flexion was observed in 33%. Back mobility was associated with body mass index (BMI), popliteal angle, and Beighton score but not sex, race or retained lordosis. The mean interincisor distance measurement was 47 mm (95% CI 35 mm to 60 mm) and was associated with height and BMI but not sex, race, or Beighton score.

**Conclusion:**

Normal values for lower back range of motion and interincisor distance were obtained which are needed in pediatric rheumatologic clinics and do not significantly vary as to race or sex. Retained lordosis on forward flexion is a normal variant. Hamstring tightness, hypermobility and BMI need to be considered when ascertaining back mobility.

## Background

Children with various forms of juvenile idiopathic arthritis are at risk for developing spinal arthritis particularly of the lower spine and subsequent restricted range of motion to the lower back. In adults, disease activity in spinal arthritides such as ankylosing spondylitis is monitored by multiple measures including radiology, and disability [1] as well as spinal mobility assessment. The latter measurement is particularly important as loss of mobility can be an early feature [2], is utilized for classification criteria [2], and loss of spinal mobility has been reported to be a poor prognostic factor [3]. In contrast, in pediatric patients, scales defining normal and abnormal range of back, popliteal angle, and jaw mobility have only been described in a few patients [4-6] or in a homogenous group of patients [7]. An English study in 1979 on 390 subjects reports a bimodal curve [5]. However, in that paper, not enough data were reported to analyze why this curve was generated. In 1986 Haley, et al., conducted a similar study on 282 children aged 5–9 years, and Siamopoulou-Mavridou reported on 393 children aged 7–14 years, but again these reports do not allow for percentiles to be generated [4,6]. Further, no author has attempted to correlate the back range of motion with hypermobility or hamstring tightness. Additionally, it might make sense that height would better correlate to lower back range of motion than age but this possible association was not assessed.

Likewise, arthritis of the temporomandibular joint (TMJ) is common but there is only one report of normal values for the TMJ or interincisor distance, measurement in 1,011 German children aged 10 to 17 years and factors such as hypermobility were not reported [8]. Therefore, in children, identifying interincisor distance abnormalities and following the disease progression are limited by the paucity of normal range of mobility data. In light of the lack of complete data, we sought to conduct a broad, random cross-sectional study of racially and ethnically diverse North American children to determine normal values and percentiles of interincisor distance and lower back flexion. In addition to defining the normal range of motion of the lower back and interincisor distance, we sought to ascertain if these ranges of motion were correlated with age, height, weight, sex, race, hypermobility or hamstring tightness. Our hypotheses were: 1) lower back range of motion is correlated more closely with height rather than age, 2) lower back range of motion is directly correlated with hamstring tightness as measured by popliteal angle, 3) tooth-to-tooth excursion is associated more closely with hypermobility rather than height or age, 4) there are no difference in these measurements between races but 5) females have greater ranges of motion compared to males of the same age, as found in other similar studies [4-8].

## Methods

### Subjects

Children aged 5 to 17 years who sought treatment in the Emergency Department during the time when one of the authors (SLW) was available to perform measurements were invited to participate. Subjects were excluded if they had a first degree relative with known ankylosing spondylitis; were obviously pregnant; in acute distress and being attended to by Emergency Department staff; had acute or chronic back pain; had other chronic illnesses that may affect joints or the back; had limb, spinal, or jaw deformities; or had acute injuries to the back, legs, or jaw. Subjects with shed incisor deciduous teeth without fully erupted permanent incisors were excluded from the interincisor distance measurement. Subject recruitment was designed to obtain equal numbers of males and females in each age range. Obviously pregnant patients were excluded only because it was felt they would not be able to bend over in a normal fashion.

### Measurements

**Figure 1:**
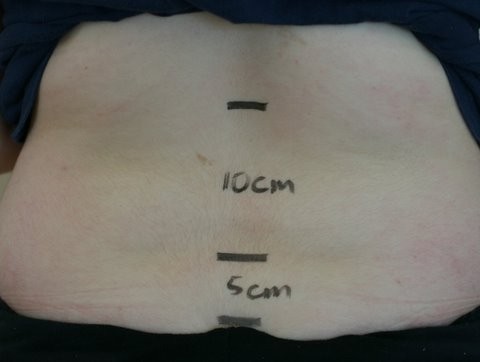
**Modified Schober Landmarks.** The first line is placed level with the superior borders of the posterior superior iliac spine (at the dimple of Venus). The other lines are then marked 10 cm above and 5 cm below it while standing erect. The value is obtained by measuring the expansion of this 15 cm segment when the subject is fully flexed forward.

Demographic data was self-reported. All measurements were performed by one of the authors (SLW). Weight was obtained by electronic scale with clothes on but without shoes. Height was measured by tape measure. The popliteal angle was measured by standard technique using a goniometer [9]. Body mass index (BMI) was calculated using standards established for subjects under the age of 18 [10]. Hypermobility was ascertained using the 9 maneuvers of the Beighton score [7]. The Schober and modified Schober measurements were obtained as defined in the literature [11,12], see Figure 1. The presence of a retained lordosis was noted as viewed from the sagital view and confirmed if the tape measure, when measuring the modified Schober, came off the skin. Interincisor distance range was determined by measuring the incisor tooth-to-tooth gap using a TheraBite ® (Atos Medical, Hörby, Sweden). All patients who were asked to participate received a small token of appreciation, such as a sticker. The study was approved by the Institutional Review Board.

### Statistical analyses

Statistical analysis was performed with Stata 10.0 (StataCorp, College Station, TX, USA). Covariates of interest were tested for association with the Schober’s, modified Schober’s, and tooth-to-tooth gap measurements by univariate linear regression and all significant associations (p <0.05) are reported. Adjustment for potential confounding was performed by building separate explanatory models for each significant covariate. These explanatory models included all covariates that confounded the association of interest, as defined by a greater than 10% change in the value of the coefficient. Only main effects were considered. Due to co-linearity, BMI was analyzed and weight was not.

## Results

### Subjects

Measurements were obtained from 307 subjects. The subjects were 51% male and 55% African-American, 29% Caucasian, 5% Latino, 5% Asian, and 6% mixed-race or other. By design, the subjects’ ages were fairly uniformly distributed between 5 and 16 years old.

### Standard measurements

**Figure 2:**
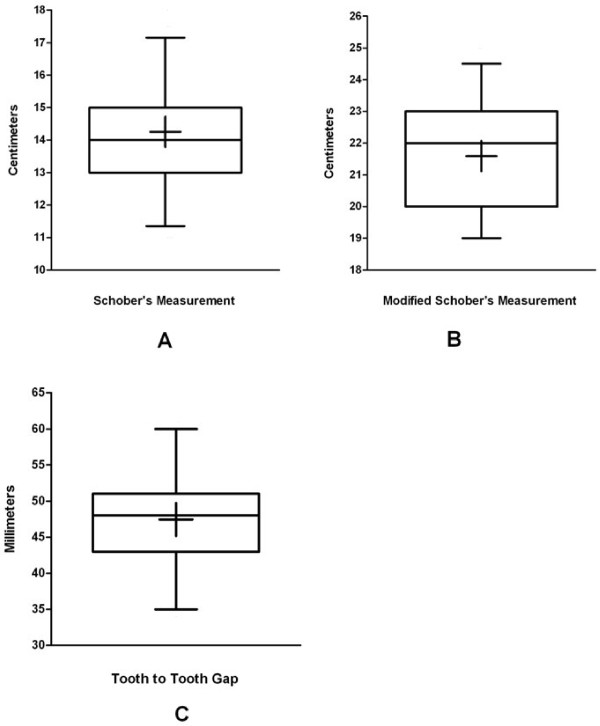
**The range of measurements obtained in healthy subjects for (A) Schober’s, (B) modified Schober’s, and (C) tooth-to-tooth gap.** The bottom and top of the box plot represent the sample 25th and 75th percentiles, respectively. The line through the box represents the sample median (50th percentile). The cross represent the sample mean. The lower and upper whiskers represent the sample 2.5 and 97.5 percentiles, respectively.

The range of measurements obtained in the healthy subjects for Schober’s, modified Schober’s, and tooth-to-tooth gap are depicted in the box plots in Figure 2. For the Schober’s measurements, the mean was 14.3 cm with a sample 95% confidence interval of 11.2 cm to 17.3 cm. The mean modified Schober’s measurement was 21.6 cm with a sample 95% confidence interval of 18.4 cm to 24.8 cm. In total, 101 (33%) of subjects demonstrated retained lumbar lordosis. For the tooth to tooth gap measurement, the mean was 47 mm with a sample 95% confidence interval of 35 mm to 60 mm. The mean popliteal angle among all subjects was 149° with a standard deviation of 8°. The median Beighton score was 2. A Beighton score of zero was found in 40% of subjects. A Beighton score of 4 or greater was found in 27% and score of 6 or greater was found in 19% of subjects.

### Predictors of measurements

In univariate linear regression analysis, 5 variables were associated with the Schober’s measurement as shown in Table 1. Not associated with Schober’s measurement were: sex, race, retained lumbar lordosis, and ability to touch palms to floor. After adjusting for confounding, BMI, popliteal angle, and Beighton score remained significantly associated with the Schober’s measurement. However, the model containing these 3 variables explained only a modest proportion of variance in the Schober’s measurements (adjusted R^2^ = 0.31).

**Table 1:** Association of variables with Schober’s measurement

**Covariate**	**Unadjusted coefficient**	**p value**	**R^2^ value**	**Adjusted coefficient**	**p value**
Age (years)	0.11 (0.059 – 0.16)	<0.001	0.057	0.025 (−0.025 – 0.076)	0.32
Height (cm)	0.017 (0.008 – 0.026)	<0.001	0.042	0.007 (−0.007 – 0.021)	0.33
BMI (kg/m2)	0.096 (0.068 – 0.12)	<0.001	0.13	0.078 (0.049 – 0.11)	<0.001
Popliteal angle (degrees)	0.085 (0.066 – 0.10)	<0.001	0.21	0.085 (0.066 – 0.10)	<0.001
Beighton score	0.091 (0.029 – 0.15)	0.004	0.027	0.082 (0.023 – 0.14)	0.007

The results for the modified Schober’s test were similar. In univariate linear regression analysis, 6 variables were associated with the modified Schober’s measurement as shown in Table 2. Not associated with the modified Schober’s measurement were: sex, race, and retained lumbar lordosis. After adjusting for confounding, height, BMI, popliteal angle, and Beighton score remained significantly associated with the modified Schober’s measurement. However, the model containing these 4 variables explained only a modest proportion of variance in the modified Schober’s measurements (adjusted R^2^ = 0.31).

**Table 2:** Association of variables with modified Schober’s measurement

**Covariate**	**Unadjusted coefficient**	**p value**	**R^2^ value**	**Adjusted coefficient**	**p value**
Age (years)	0.15 (0.097 – 0.20)	<0.001	0.096	-0.025 (−0.11 – 0.59)	0.56
Height (cm)	0.028 (0.019 – 0.038	<0.001	0.11	0.023 (0.008 – 0.038)	0.004
BMI (kg/m2)	0.12 (0.086 – 0.14)	<0.001	0.17	0.094 (0.061 – 0.13)	<0.001
Popliteal angle (degrees)	0.072 (0.052 – 0.093)	<0.001	0.14	0.060 (0.041 – 0.80)	<0.001
Beighton score	0.089 (0.023 – 0.15)	0.008	0.023	0.11 (0.034 – 0.19)	0.005
Touch palms to floor	0.39 (0.0005 – 0.77)	0.05	0.0007	0.094 (−0.31 – 0.50)	0.65

In univariate linear regression analysis, 4 variables were associated with the tooth-to-tooth gap measurement as shown in Table 3. Not associated with this measurement were: sex, race, and Beighton score. After adjusting for confounding, only height and BMI were associated with tooth-to-tooth gap. However, the model containing these 2 variables explained only a small proportion of the variance in the tooth-to-tooth gap measurements (adjusted R^2^ = 0.20).

**Table 3:** Association of variables with tooth-to-tooth gap measurement

**Covariate**	**Unadjusted coefficient**	**p value**	**R^2^ value**	**Adjusted coefficient**	**p value**
Age	0.73 (0.54 – 0.92)	<0.001	0.15	0.20 (−0.15 – 0.55)	0.26
Height (cm)	0.14 (0.11 – 0.18)	<0.001	0.17	0.089 (0.029 – 0.15)	0.004
BMI (kg/m2)	0.33 (0.21 – 0.45)	<0.001	0.093	0.14 (0.017 – 0.27	0.026
Permanent teeth	3.8 (2.3 – 5.2)	<0.001	0.080	0.47 (−1.2 – 2.2)	0.58

There was no difference between males and females for BMI, height, weight, popliteal angle, Schober, Modified Schober, tooth-to-tooth gap and Beighton score.

## Discussion

The aim of this study was to generate normal values of lower back mobility, and interincisor distance and popliteal range of motion for a racially and age range diverse group of children. The results gathered showed three significant findings.

Back mobility as measured by the Schober and Modified Schober methods best correlated to hamstring tightness as measured by the popliteal angle, Beighton score and BMI. It makes sense that the more flexible you are the more flexible is your lower back and the tighter one’s hamstrings are the more restricted is one’s back mobility. Likewise with an increasing BMI the arc of the lower back is greater since the skin is farther away from the vertebral bodies.

When measuring lower back mobility, we are struck by the number of children who do not fully reverse their lumbar lordosis. This is, however, not uncommon in the normal population and should not be considered abnormal.

Jaw mobility, however, was not correlated to overall flexibility as we hypothesized but rather more closely related to height and BMI. A tooth to tooth measurement under 3.5 cm should be considered abnormal.

It is reassuring that there was no difference in these measurements based on race or sex. At one time ankylosing spondylitis was considered an affliction of Caucasian males but there is an increased awareness of this condition in females and minorities [3,13,14]. Since these conditions frequently present in childhood, we can be confident that the numbers we generated can be applied to a diverse population.

A limitation of this study was the manner in which some of the data was collected. We designed data collection methods so as to ensure that they were simple and easily reproducible in most settings. Therefore, we asked for the patient to self-report race and weight, with the assumption that weight was recently assessed by the emergency department staff. In addition, height was obtained by a tape measure, not a standard stadiometer. It is our hope that we did not sacrifice accuracy for ease.

We believe the methods used were objective and simple enough to be reproducible and based upon evidence gathered by other studies [5,8,11,15]. Particularly for children, few studies exist which provide any objective criteria for the epidemiological diagnosis of spondyloarthropathy. The present study offers normal values for mobility which are needed in pediatric rheumatology clinics. A modified Schober of less than 18 cm is abnormal. In the future, a second study should repeat the same protocol with a pediatric population with known spondyloarthropathy. The results should be compared with results from this study to offer percentages of difference of mobility from normal that patients with spondyloarthropathy have.

## Conclusion

Normal values for lower back range of motion and tooth-to-tooth gap was obtained on 307 children. Schober under 13 cm and Modified Schober under 20 cm are 1 Standard Deviation below normal. These measurements are reduced if the hamstrings are tight and increased if the BMI is high. Tooth-to-tooth gap was increased in relationship to height but not to hypermobility and should be greater than 42 mm (1 standard deviation). Age, race and sex were not significant factors in our results.

## Abbreviations

TMJ, Temporomandibular joint; BMI, Body mass index.

## Competing interest

The authors, Sophie L. Woolston, Timothy Beukelman and David D. Sherry, do not have any financial arrangement with any company whose product figures prominently in the submitted manuscript or with a company making a competing product.

## Authors’ contributions

SLW drafted the manuscript and carried out the measurements. TB performed the statistical analyses and helped with the study design. DDS formulated the study design. All authors critically contributed to, reviewed and revised drafts of the manuscript. All authors read and approved the final manuscript.

## Funding

Funding was obtained through the American College of Rheumatology Research and Education Foundation (REF) Medical Student Research Preceptorship (MSRP) Award.
